# Structural and functional analysis of the *Helicobacter pylori* lipoprotein chaperone LolA

**DOI:** 10.3389/fmicb.2024.1512451

**Published:** 2024-12-19

**Authors:** Deepika Jaiman, Karina Persson

**Affiliations:** ^1^Umeå Centre for Microbial Research (UCMR), Umeå University, Umeå, Sweden; ^2^Department of Chemistry, Umeå University, Umeå, Sweden

**Keywords:** lipoprotein transport, LolA, polymyxin, ITC, crystal structure, ε-proteobacteria

## Abstract

Lipoproteins are crucial for maintaining the structural integrity of bacterial membranes. In Gram-negative bacteria, the localization of lipoprotein (Lol) system facilitates the transport of these proteins from the inner membrane to the outer membrane. In *Helicobacter pylori*, an ε-proteobacterium, lipoprotein transport differs significantly from the canonical and well-studied system in *Escherichia coli*, particularly due to the absence of LolB and the use of a LolF homodimer instead of the LolCE heterodimer. This study presents the crystal structure of the *H. pylori* lipoprotein chaperone LolA (LolA-HP) and its interaction with lipopeptide antibiotics such as polymyxin B and colistin. Isothermal titration calorimetry revealed that, unlike LolA from *Vibrio cholerae* and *Porphyromonas gingivalis*, LolA-HP does not bind to these antibiotics. Structural comparisons showed that LolA-HP has a deeper hydrophobic cleft but lacks the negative electrostatic potential critical for binding polymyxins. These findings offer insights into the structural diversity of LolA across bacterial species and its potential as a target for antibacterial agents.

## Introduction

**Figure 1:**
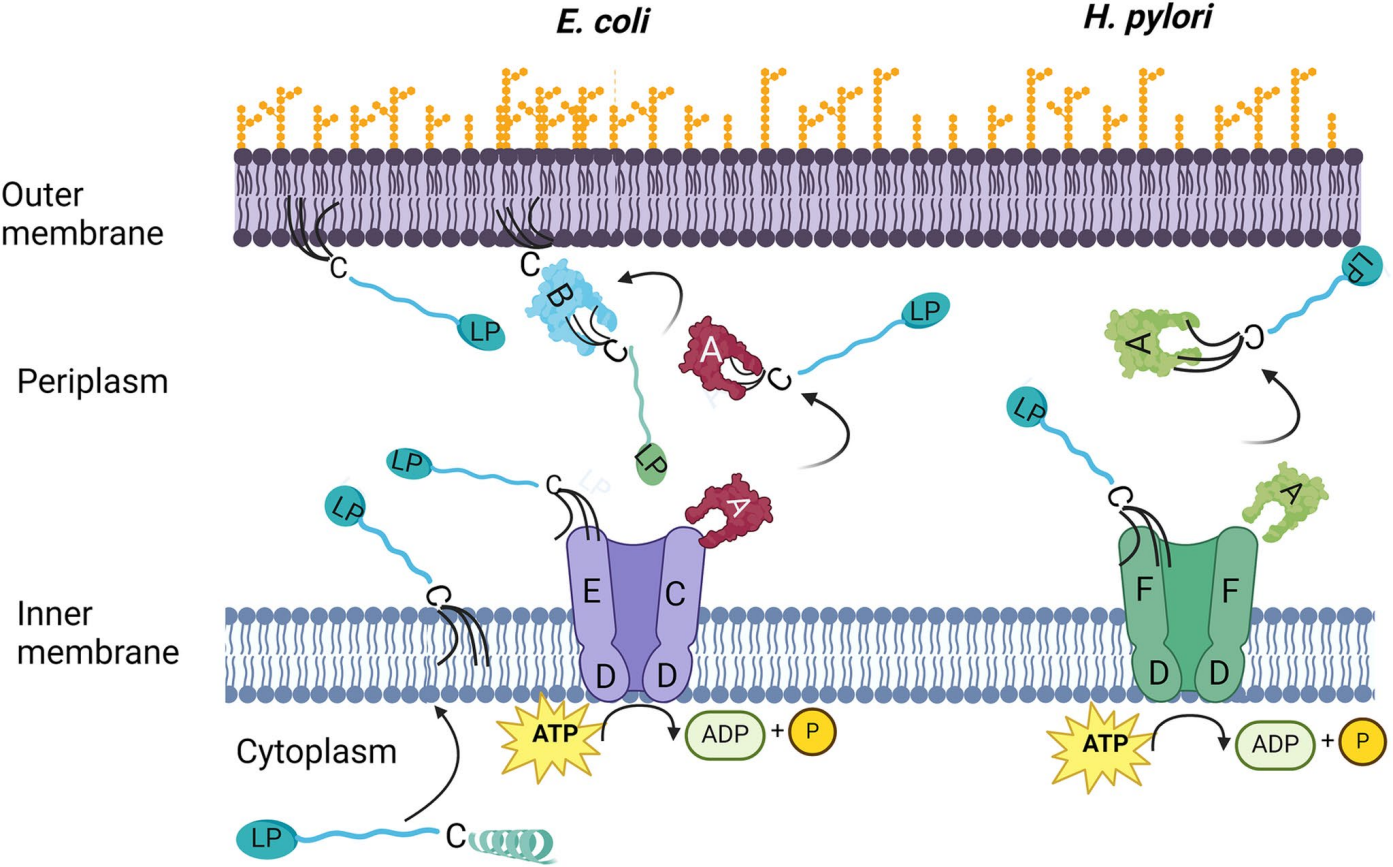
Lipidation and transport of lipoproteins. The prelipoprotein is transported over the IM and the signal peptide is removed. Three acyl chains are added to the N-terminal cysteine which anchors the protein to the membrane. In *E. coli* (left), the lipoprotein is recognized by the LolCED_2_ which transfers it to the chaperone protein LolA. Next, the lipoprotein will be delivered to LolB, which in turn will transfer it to the OM. In *H. pylori* (right) the LolCE heterodimer is exchanged for a LolF_2_ dimer. A protein corresponding to LolB has not been found in *H. pylori*. The figure was created with BioRender.com.

Gram-negative bacteria are characterized by a unique double-membrane structure consisting of an inner membrane (IM) and an outer membrane (OM), with a periplasmic space enriched with peptidoglycans between them. Both membranes are lipid bilayers in which a wide variety of associated and integral membrane proteins are embedded. Among the OM proteins, porins, structured as β-barrels, facilitate the passive transport of molecules across the membrane (Rollauer et al., 2015). The outer leaflet of the OM is mainly composed of glycolipids and lipopolysaccharides, which serve as a protective barrier and communication interface for the bacteria, whereas the inner leaflet consists of phospholipids. In contrast, both leaflets of the IM consist of phospholipids, and the proteins that span the IM predominantly adopt helical structures, performing critical functions such as translocating proteins from the cytoplasm (Orfanoudaki and Economou, 2014; Green and Mecsas, 2016), mediating metabolite exchange, and driving the electron transport chain to generate energy (Wallin and von Heijne, 1998). Both membranes also contain lipoproteins, which are synthesized as pre-lipoproteins in the cytoplasm, transported over the IM (Green and Mecsas, 2016), and anchored to the lipid bilayer through acyl chains covalently attached to a conserved cysteine residue. This anchoring process involves several enzymatic steps: diacylglycerol transferase (Lgt) catalyzes the attachment of a diacylglyceryl to the sulfhydryl of the cysteine (Sankaran and Wu, 1994) resulting in a diacylated protein, and signal peptidase II (LspA) removes the signal peptide, exposing the acylated cysteine as the first residue (Vogeley et al., 2016). In many bacteria, such as *Escherichia coli*, the enzyme apolipoprotein N-acyltransferase (Lnt) further acylates the free amino group of the cysteine, resulting in a fully matured lipoprotein with three acyl chains (Hillmann et al., 2011). The fate of the lipoprotein, whether retained in the IM or transported to the OM, depends on a sorting motif after the acylated cysteine. In the case of OM-targeted lipoproteins, the localization of lipoprotein (Lol) system plays a key role (Narita et al., 2004; Okuda and Tokuda, 2011). This system, particularly well-studied in *E. coli*, consists of the ABC transporter complex, LolCDE. In this complex, LolE extracts the lipoprotein from the IM and transfers it to the chaperone LolA, which is bound to the periplasmic domain of LolC. A dimer of LolD is located on the cytoplasmic side of the LolCE dimer and provides energy to this process via its ATPase activity (Figure 1). LolA then forms a complex with the lipoprotein, transports it across the periplasm and delivers it to LolB, a recipient protein, which itself is a lipoprotein, anchored to the OM. In the next step, LolB transfers the acyl chains of the lipoprotein to the inner leaflet of the OM (Narita et al., 2004; Okuda and Tokuda, 2011). Notably, LolB has only been identified in β- and γ-proteobacteria and appears to be absent in all other bacterial classes. In the α-proteobacteria, *Caulobacter vibrioides*, LolA (LolA-CV) has been shown to perform both transport and insertion of lipoproteins into the OM (Smith et al., 2023).

*Helicobacter pylori* is an ε-proteobacterium known for colonizing the human gastric mucosa and for contributing to conditions such as peptic ulcers and gastric inflammation (Suerbaum and Michetti, 2002; Nomura et al., 2002). *H. pylori* harbors approximately 40 lipoproteins, including CagT, a component of the type IV secretion system that secretes the oncoprotein CagA (Babu et al., 2006; Backert et al., 2015). Despite its reliance on functional lipoprotein transport, the Lol system in *H. pylori* differs significantly from the canonical system in *E. coli*. Specifically, *H. pylori* lacks the LolB receptor protein at the OM and, instead of the LolCE dimer at the IM utilizes a homodimeric LolF (Figure 1), which combines the functions of LolC and LolE (LoVullo et al., 2015). This divergence underscores the critical role of acylation and transport in maintaining bacterial membrane integrity, making these processes attractive targets for antibacterial drug development.

Inhibiting components of the Lol system, particularly LolCDE in *E. coli*, has been explored as a potential strategy for novel antimicrobial agents (Nickerson et al., 2018; Caro et al., 2019). Additionally, LolA has been implicated in the transport of polymyxins—lipopeptide antibiotics used as a last-resort treatment—across the periplasm to the IM (Weerakoon et al., 2021; Pedebos et al., 2021). Previous studies have shown that LolA from bacteria such as *Porphyromonas gingivalis* and *Vibrio cholerae* bind polymyxin B, while LolB from *V. cholerae* does not (Jaiman et al., 2023). To assess the potential of LolA as a broad-spectrum antibacterial target, it is essential to investigate its structure and binding properties across different bacterial classes. If LolA exhibits conserved binding mechanisms, it could serve as a universal target for Gram-negative bacteria. However, structural variability might limit its applicability to specific bacterial species. While crystal structures of LolA have been determined for several γ-proteobacteria, including *Pseudomonas aeruginosa*, *E. coli*, *Yersinia pestis*, and *V. cholerae* (Jaiman et al., 2023; Remans et al., 2010; Takeda et al., 2003), as well as the Bacteroidota *P. gingivalis* (Jaiman et al., 2023), structural studies on other bacterial classes remain limited. Additionally, LolA from *E. coli* (LolA-EC) has been studied in complex with LolC both using crystallography and cryo-electron microscopy (Kaplan et al., 2018; Tang et al., 2021). These studies were recently supplemented with the crystal structure of LolA-EC in complex with a triacylated peptide, offering valuable insights into complex formation and function (Kaplan et al., 2022). The recent determination of a LolA-LolB complex in *Xanthomonas campestris* (γ-proteobacteria) (Furlanetto and Divne, 2023) further highlights the diversity of the Lol system. In this study, we present the crystal structure of LolA from *H. pylori* (LolA-HP) and examine its interaction with lipopeptide antibiotics. Our findings provide a foundation for future drug discovery efforts targeting the Lol system across diverse classes of Gram-negative bacteria.

## Materials and methods

### Cloning, overexpression, and purification of LolA

The *lolA* gene from *H. pylori* strain J99 (GenBank AAD06296) was PCR amplified from genomic DNA (primer sequences are presented in Supplementary Table S1). Primers were designed not to include the signal peptide residues 1–19. The *lolA* PCR product was digested with *NcoI*/*EcoRI* and ligated into equivalent sites of pET-His1a, in-frame with a tag with sequence MKHHHHHHPMSDYDIPTTENLYFQGAM followed by LolA residues 20–184. The protein was overexpressed in *E. coli* BL21 (DE3) cells in LB medium at 37°C and induced with 0.5 mM isopropyl β-d-1-thiogalactopyranoside (IPTG). The cells were harvested by centrifugation, and the pellets were stored at −80°C until further use. The cell pellets were resuspended in buffer (50 mM sodium phosphate pH 7.6, 0.3 M NaCl) containing 10 mM imidazole and supplemented with 1% Triton X-100. After sonication on ice, the lysate was centrifuged (63,000×*g* for 20 min). The supernatant was incubated with His60 Ni-resin (Takara Bio). The Ni-resin was washed with the same buffer containing 30 mM imidazole and transferred to an Econo-Pac column (Bio-Rad), from which the protein was eluted with a buffer containing 0.3 M imidazole. The histidine tag was removed by incubation with ~1% (w/w) TEV protease overnight at +4°C at reducing conditions. The buffer was exchanged (50 mM sodium phosphate pH 7.6, 0.2 M NaCl), and the protein was passed over the Ni column again. The flow-through fractions were concentrated and further purified by gel filtration (HiLoadTM 16/60 Superdex™200 prep-grade column (GE Healthcare)) equilibrated with 20 mM Tris pH 7.5, 150 mM NaCl. LolA from *P. gingivalis* and *V. cholerae* were purified as described previously (Jaiman et al., 2023). The purity of the proteins is shown in Supplementary Figure S3.

### Crystallization and structure determination

LolA was screened for crystals with crystallization screens from Molecular Dimensions using sitting-drop vapor diffusion at room temperature in 96-well MRC-crystallization plates (Molecular Dimensions). Droplets of 0.2 μL protein were mixed with 0.1 μL of mother liquor using a Mosquito (TTP Labtech) pipetting robot. The protein concentration was 105 mg/mL. Crystals were obtained from the MIDAS Plus screen in several conditions. The crystal used for structure determination was grown in 50% pentaerythritol propoxylate and 0.1 M Tris pH 8, flash-cooled in liquid nitrogen, and stored until data collection. Diffraction data were collected remotely at 100 K on beamline ID30B operated by the European Synchrotron Radiation Facility (ESRF), Grenoble, France. The diffraction images were automatically processed with XDS (Kabsch, 2010), and data reduction was performed using Aimless (Evans and Murshudov, 2013) from the CCP4 package (Winn et al., 2011). The structure of LolA-HP was solved by molecular replacement using Phaser (McCoy, 2007) and a search model produced by AlphaFold (Jumper et al., 2021). Rounds of refinement and model building were performed using phenix.refine (Afonine et al., 2012) and COOT (Emsley et al., 2010). Figures were prepared using CCP4MG (Schrodinger, LLC, 2015; McNicholas et al., 2011) and BioRender. Data processing and refinement statistics are presented in Supplementary Table S2.

### Sequence alignments

The protein sequences of LolA from *H. pylori* (WP_000643052), *E. coli* (WP_247094763), *V. cholerae* (EHU6507077), *P. gingivalis* (WP_012457548), *X. campestris* (WP_000643052), and *C. vibrioides* (WP_096034663) were pairwise aligned using Clustal Omega (Madeira et al., 2024) to calculate the percentage similarity. All sequences were further aligned with T-Coffee (Floden et al., 2016) and visualized with Espript3 (Robert and Gouet, 2014).

### Isothermal titration calorimetry experiments

Isothermal titration calorimetry (ITC) experiments were conducted as described previously (Jaiman et al., 2023). In short, the experiments were performed at 25°C using a MicroCal auto-iTC200 instrument in a buffer containing 20 mM HEPES pH 7.5 and 150 mM NaCl. For interactions involving LolA-HP, LolA-VC, LolA-PG, and polymyxin B/colistin/nonapeptide, the stirring speed was maintained at 1000 rpm.

Control experiments were performed for both the proteins and polymyxin derivatives by injecting ligands at the same concentrations used in the tests into the buffer. The resulting data were subtracted from the corresponding interaction data using a linear fit. Raw data were analyzed with the MicroCal PEAQ-ITC software, applying a single-site binding model for all datasets. Polymyxin B, colistin, and nonapeptide were obtained from Merck.

## Results

### Overall architecture of LolA from *H. pylori* and comparison with other LolA proteins

LolA from *H. pylori* was overexpressed, and the protein was purified and crystallized. After X-ray diffraction data were collected, the structure of LolA-HP was determined with molecular replacement using a search model obtained from AlphaFold (Jumper et al., 2021). The structure was refined to 2.05 Å with final R_work_ and R_free_ values of 20.2 and 25.2%, respectively (Supplementary Table S2).

**Figure 2:**
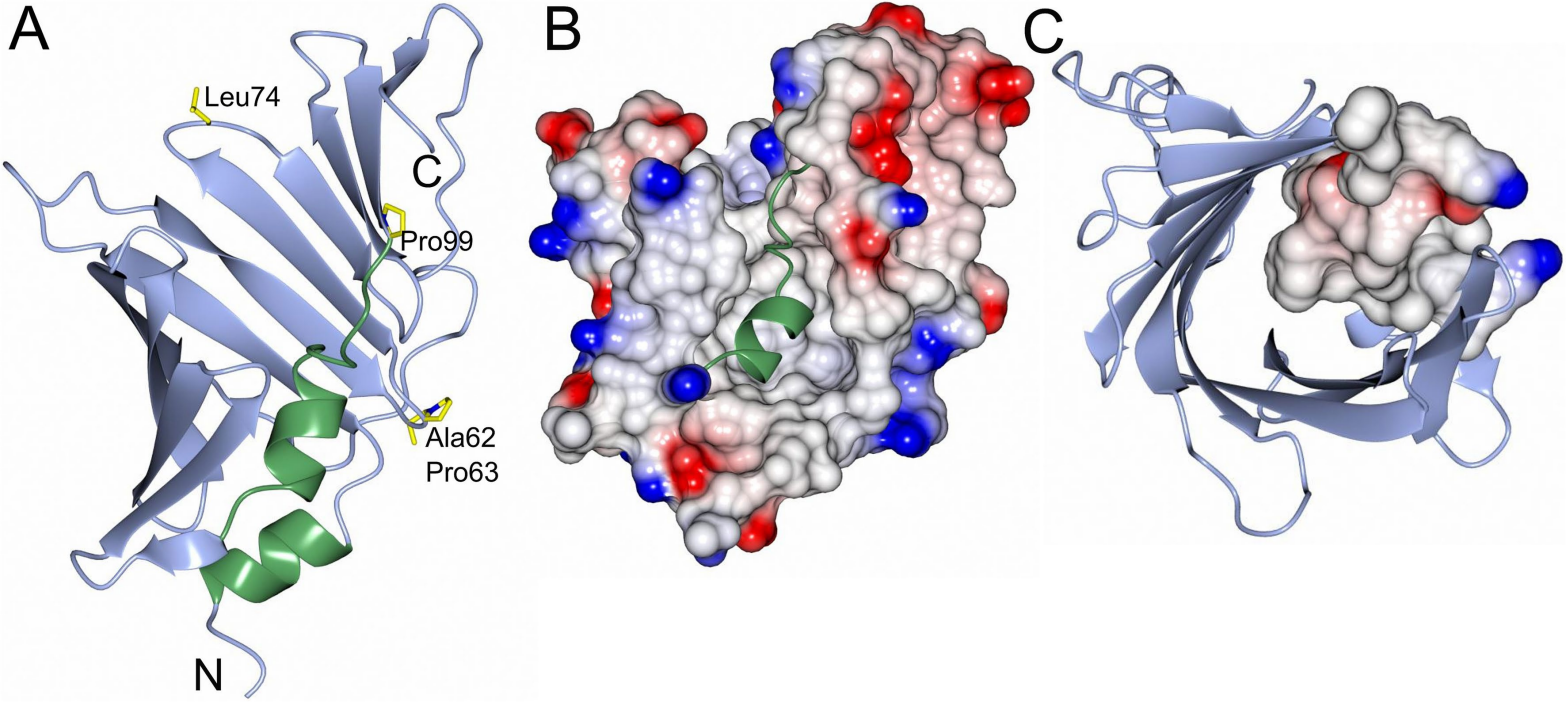
Overall structure and electrostatic map of LolA-HP. **(A)** LolA from *H. pylori* with the open β-barrel depicted in blue and the helices and turns that fill the cavity in green. Residues that may be of interest for function (Ala62, Pro63, Leu74, and Pro99) are depicted as cylinders and labeled. **(B)** Electrostatic surface representation of LolA-HP where the helix and turns in the cavity are shown as ribbons in green. **(C)** LolA-HP where the structural elements filling the cavity are depicted as an electrostatic surface. The figure was created with BioRender.

**Figure 3:**
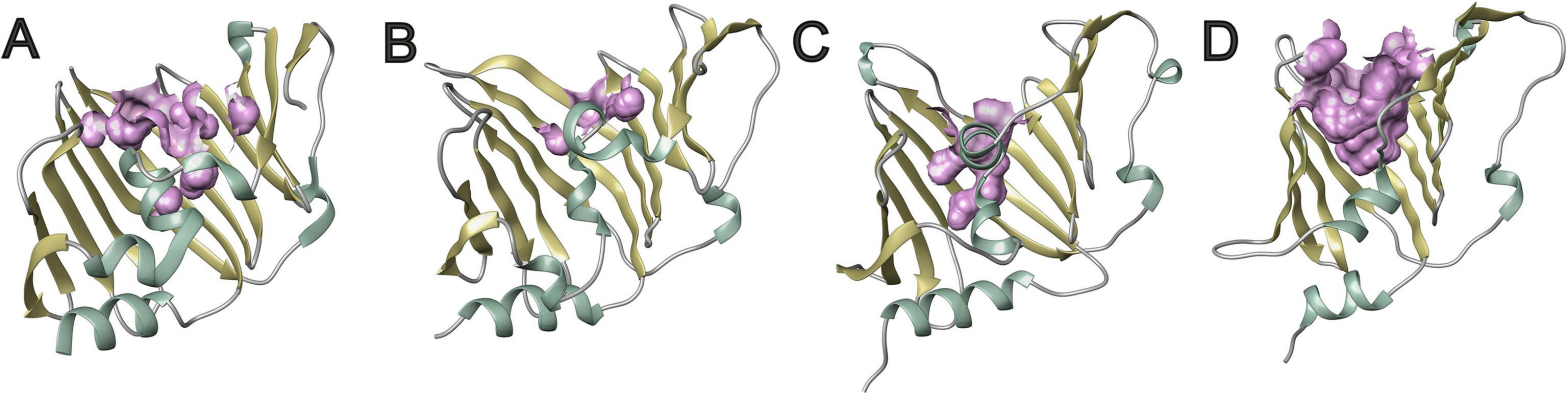
Solvent accessibility of LolA from different bacteria. **(A)** LolA-EC, **(B)** LolA-VC, **(C)** LolA-PG, and **(D)** LolA-HP. The solvent-accessible part of the binding clefts is calculated with CASTp (Tian et al., 2018) using protein structures 1IWL, 8CHX, 8CGM, and 9GTX. The figure was created with BioRender.

Despite low sequence similarities comparing LolA-HP with other LolA proteins—ranging from 26% comparing LolA-HP and LolA-EC to 19% comparing LolA-HP and LolA-PG (Table 1)—the overall structures are more similar with a root mean square deviation (RMSD) of 2.92 Å for the LolA-HP-LolA-PG pair. Similar to other LolA structures, LolA-HP comprises a curved antiparallel β-sheet that consists of 12 strands (Figure 2). The last strand, β12, links to β11 through an elongated segment that extends along the outwardly curved surface of the β-sheet. The concave side of the sheet forms a hydrophobic cleft, shielded from the adjacent solvent by the presence of a helix (α2) and an extended loop region between β6 and α2. The base of this cleft is formed by the N-terminal helix, α1. In LolA-EC and other studied LolA structures, there is an additional helix that protects the cleft, which is equivalent to the extended β6α3 loop in LolA-HP. The LolA structures studied so far, have a conserved proline in connection to the helices in the cleft which is likely to restrict their flexibility. In LolA-HP there is a proline (P99) at the end of β6 that precedes the extended region, but no other prolines are present in the segment that fills the cleft (Supplementary Figure S1). This results in a more accessible and deep binding cleft, 709 Å^3^ compared to 36 Å^3^ in LolA-PG (PDB 8CGM) (Jaiman et al., 2023), the very shallow clefts of 12 Å^3^ in LolA-EC (PDB 1IWL) (Takeda et al., 2003), and 0.15 Å^3^ in LolA-VC (PDB 8CHX) (Figure 3 and Supplementary Figure S7). The volumes were calculated with CastP (Tian et al., 2018). There is unmodeled electron density in the LolA-HP binding cleft (Supplementary Figure S4) that we hypothesize belongs to the crystallization precipitant pentaerythritol propoxylate.

**Table 1:** Pairwise sequence alignments.

Sequence identity %	LolA-EC (1IWL)	LolA-VC (8CHX)	LolA-HP (9GTX)	LolA-PG (8CGM)	LolA-XC (8ORN)	LolA-CV (model)
LolA-EC (1IWL)	100	39	26	22	26	18
LolA-VC (8CHX)		100	24	26	31	23
LolA-HP (9GTX)			100	19	22	22
LolA-PG (8CGM)				100	25	24
LolA-XC (8ORN)					100	23
LolA-CV (model)						100

The protein sequences of LolA from *E. coli* (EC), *V. cholerae* (VC), *H. pylori* (HP), *P. gingivalis* (PG), *X. campestris* (XC), and *C. vibrioides* (CV) were pairwise aligned using clustal omega. The PDB codes are shown when applicable.

### Conserved residues in LolA and LolB

The sequence similarity between LolA from different bacteria is generally low but highly conserved within different *H. pylori* strains (Table 1 and Supplementary Figures 1, 2). In our comparison, LolA-EC and LolA-VC, which share 39% sequence similarity, are the most similar. Both are γ-proteobacteria and share an Arg-Pro motif, located on the β2β3 loop, which, in *E. coli*, has been suggested to be important for lipoprotein binding and delivery to LolB (Kaplan et al., 2022). In contrast, LolA-HP, which is reported not to have a LolB receptor instead features an Ala-Pro sequence in the corresponding loop. This motif resembles LolA from *X. campestris*, (LolA-XC), a γ-proteobacterium, which has a Thr-Pro at the same position. Notably, LolA-XC has a LolB partner to which it binds effectively despite the absence of the arginine residue (Furlanetto and Divne, 2023). Studies on LolB from *E. coli* (LolB-EC), have shown that a conserved leucine that is exposed on the loop that connects strands β3 and β4 is crucial for lipoprotein delivery to the OM (Hayashi et al., 2014). This leucine is also found in the same position in LolB-VC; however, LolB-XC instead has a valine in the same position. The extensive sequence variability across species and the lack of conservation in motifs suggest that generalizations about specific residues being essential for certain functions may not be valid.

### Binding to polymyxin B, colistin, and nonapeptide

Polymyxins are cyclic cationic lipopeptide antibiotics that disrupt bacterial membranes through electrostatic interactions with lipid A. It has been proposed that LolA transports the lipid tail of polymyxin across the periplasm to the IM (Weerakoon et al., 2021; Pedebos et al., 2021). Previous ITC studies showed that LolA-VC and LolA-PG bind polymyxin B with dissociation constants (Kd) of 56 μM and 14 μM, respectively, while LolB-VC binds very poorly (Jaiman et al., 2023). In this study, the experiments were repeated to also include colistin, nonapeptide (polymyxin B without acyl chain) (Supplementary Figure S5), and LolA-HP. The dissociation constants are shown in Table 2 and Supplementary Figure S6. In contrast to LolA-VC and Unlike LolA-VC and LolA-PG, LolA-HP showed only non-reproducible results, indicating no or very weak binding to all tested compounds.

**Table 2:** ITC data for colistin, polymyxin, nonapeptide, and LolA proteins.

Protein	Kd (Colistin)	Kd (Polymyxin B)	Kd (Nonapeptide)
LolA-PG	33 μM	14 μM	Weak
LolA-VC	91 μM	56 μM	Weak
LolA-HP	No binding	No binding	No binding

**Figure 4:**
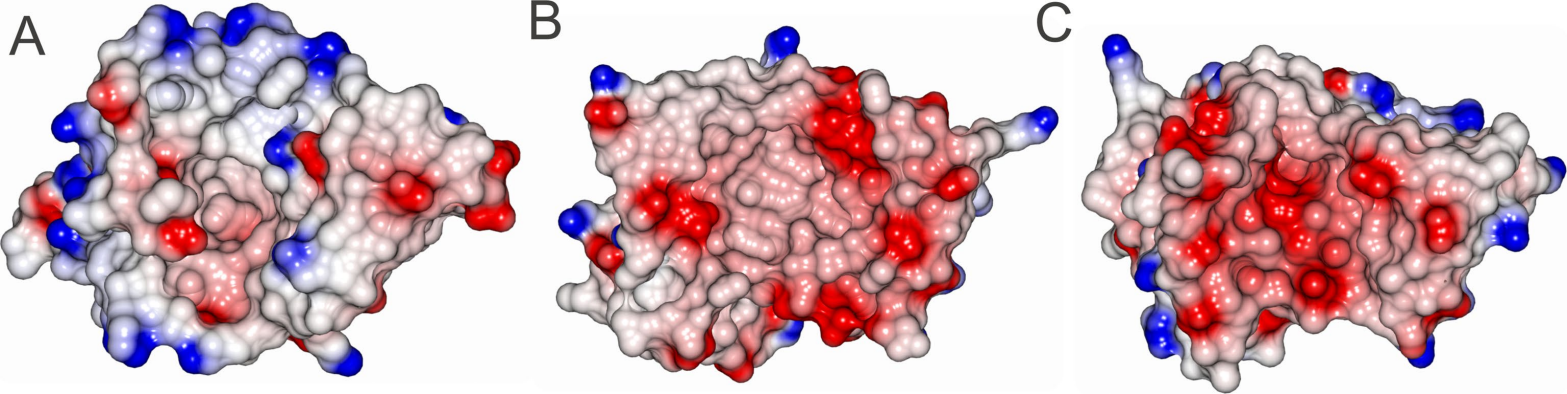
Electrostatic representation of the opening of the hydrophobic cavity of LolA from different bacteria. **(A)** LolA-HP, no binding to polymyxins, **(B)** LolA-VC binds polymyxin with a dissociation constant of 56 μM, and **(C)** LolA-PG binds polymyxin with a dissociation constant of 14 μM. The figure was created with BioRender.

Analysis of the electrostatic properties of LolA-PG, LolA-VC, and LolA-HP illustrated that LolA-PG exhibits the most pronounced negative electrostatic potential at the mouth of the binding cleft, followed by LolA-VC with moderate negative potential, and LolA-HP, which primarily contains neutral residues (Figure 4 and Supplementary Figure S7). This variation in electrostatic potential may account for the observed differences in affinity for the positively charged substances, where LolA-PG has a higher affinity for both colistin and polymyxin B compared to LolA-VC whereas LolA-HP exhibits no binding. Neither of the tested proteins shows strong interaction with nonapeptide.

## Discussion

*Helicobacter pylori* belongs to the class ε-proteobacteria, and its Lol machinery exhibits significant differences compared to the well-characterized system in *E. coli*, which represents the class γ-proteobacteria. In *E. coli*, the IM complex LolCDE plays a crucial role: LolE extracts lipoproteins from the membrane, while LolC transfers them to LolA. Conversely, *H. pylori* utilizes a LolF dimer that integrates both of these functions (LoVullo et al., 2015; McClain et al., 2020). In both organisms, a LolD dimer located on the cytoplasmic side provides energy through its ATPase activity.

Additionally, *E. coli* and other γ-proteobacteria, feature LolB, a lipoprotein anchored to the inner leaflet of the OM, which receives the lipoprotein cargo from LolA. In this transfer, LolA and LolB are hypothesized to interact through a mouth-to-mouth mechanism, driven by electrostatic attraction between the negatively charged LolA and the positively charged LolB, during which the acyl groups of the lipoprotein bound to LolA slide to the binding cleft of LolB (Okuda and Tokuda, 2009). Subsequently, by an unknown mechanism, the acyl chains are transferred from LolB to the lipid bilayer resulting in the anchoring of the lipoprotein to the membrane. It has been shown that an exposed and conserved Leu in the loop between β3 and β4 in LolB-EC is critical for its ability to anchor lipoproteins (Hayashi et al., 2014). This Leu is not completely conserved in all γ-proteobacteria; instead, *X. campestris* has a valine in the same position and the crystal structure of its LolA-LolB complex clearly shows that the loop containing the valine is deeply buried in the binding cleft of LolA, in close contact with many hydrophobic residues. Interestingly, a modeled structure of LolA from the α-proteobacteria *C. vibrioides*, (LolA-CV), a bacterium that does not have a LolB protein, also shows an exposed leucine, but in a long loop connecting strands β8 and β9. It has been demonstrated that LolA-CV can compensate for the functions of both LolA and LolB in *E. coli* Δ*lolA* and Δ*lolB* deletion mutants and that this leucine is particularly important (Smith et al., 2023). Hence, LolA-CV has dual roles which indicates that dual functionality is a possible solution for lipoprotein membrane association in bacteria that lack the LolB protein. *H. pylori* does not have a LolB and indeed LolA-HP intriguingly possesses a leucine (Leu74), in the same position as LolB-EC, suggesting that LolA-HP also might fulfill a dual function. However, this calls for a more extensive study as leucines are present on several loops in LolA-HP: Leu46 on the β1β2 loop, Leu74 on the β3β4 loop, and Leu92 on loop β5β6.

An alternative hypothesis regarding lipoprotein insertion into the membrane is that certain bacterial species may have evolved other proteins with the capacity to receive lipoproteins at the OM, yet these proteins are too divergent in sequence from the reference protein LolB-EC to be detected via conventional sequence analysis. This could represent an instance of convergent evolution, where proteins with different structures result in the same outcome. For example, *Bacteroides fragilis*, which lacks the Lnt enzyme, nonetheless exhibits triacylated lipoproteins. In this case, an enzyme named lipoprotein *N*-acyltransferase in *Bacteroides* (Lnb) was recently discovered and characterized, displaying similar catalytic activity as Lnt despite a different predicted structure (Armbruster et al., 2024). Further investigation is required to explore these alternatives in greater detail.

In the LolA protein of *E. coli*, an Arg-Pro motif located between β2 and β3 has been extensively analyzed. The arginine residue within this motif, which faces the base of the binding cleft, is critical for both the binding of the triacylated lipoproteins and their delivery to LolB. Sequence analysis (Supplementary Figure S1) shows that the Arg-Pro motif is relatively conserved in β-and γ-proteobacteria and is suggested to be associated with the presence of a LolB protein for interaction. Again, *X. campestris* is an exception as it has a Thr-Pro in the equivalent position. Despite these differences, LolA and LolB in *X. campestris* interact with a similar dissociation constant (Kd) as the *E. coli* LolA-LolB pair (14.6 μM vs. 30.6 μM) and is the only LolA–LolB pair that has been co-crystallized (Furlanetto and Divne, 2023; Nakada et al., 2009).

In LolA-HP, this motif is replaced by an Ala-Pro motif (Ala62, Pro63), and the space that would be occupied by the arginine side chain in LolA-EC is instead filled by a phenylalanine side chain (Phe107) on the α3-helix. Intriguingly, LolA-CV, which has been reported to have dual LolA–LolB functions has a classical Arg-Pro motif. The variation does however indicate that the sequence conservation is not strictly maintained even within the same phylum and that the Lol system is very variable.

Lol proteins are considered promising drug targets because the transport of lipoproteins is vital for the biogenesis of the membrane and the subsequent survival of Gram-negative bacteria. The main focus has been on targeting the IM protein complex LolCDE. The initial hits pyridinepyrazole (Breidenstein et al., 2024) and pyridineimidazole (Nayar et al., 2015; McLeod et al., 2015) have recently been developed into a promising substance, lolamicin, that selectively spares the gut bacteria (Muñoz et al., 2024). The soluble LolA was predicted to bind and transport acylated peptides such as the antibiotic polymyxin (Weerakoon et al., 2021; Pedebos et al., 2021), and we could indeed show experimentally that both LolA-VC and LolA-PG bind the substance (Jaiman et al., 2023). In the present study, using ITC, we demonstrated that LolA-VC and LolA-PG also bind the lipopeptide colistin with dissociation constants in the micromolar range. They also exhibit weak binding to nonapeptide, which has the same peptide backbone as polymyxin B but lacks the acyl tail. Hence the acyl part of the polymyxins is most important for binding to LolA but also the peptide part contributes to binding (Table 2).

However, binding is not a universal trait for all LolA proteins as LolA-HP does not bind polymyxin B, colistin, or nonapeptide. Comparative analysis of the electrostatic surface potentials of various LolA proteins reveals notable variability. For instance, LolA from *E. coli*, *V. cholerae*, and *P. gingivalis* mainly display negative electrostatic potential around the mouth of the binding cleft. In contrast, LolA from *H. pylori* and the modeled structure of LolA from *C. vibrioides* predominantly feature a more hydrophobic, non-polar environment around the binding cleft mouth. This observation aligns with the fact that the tested molecules carry a positive charge, which would preferentially interact with the negatively charged surfaces of LolA-VC and LolA-PG. This difference in electrostatic properties suggests that the binding affinities of LolA proteins are influenced by their surface potentials, which could be exploited in the design of selective antibacterial agents targeting specific Gram-negative pathogens (Figure 4 and Supplementary Figure S7).

Lol proteins are regarded as promising drug targets due to their critical role in lipoprotein transport, which is essential for membrane biogenesis and the survival of Gram-negative bacteria. Despite the low sequence similarity, the high structural conservation of LolA across various bacterial species (Table 1) highlights the potential for designing antibacterial agents that selectively target LolA components in specific pathogens. This approach, similar to the selective binding of lolamicin to pathogenic bacteria, could minimize the impact on commensal organisms (Muñoz et al., 2024). This highlights the importance of studying a wide variety of bacteria to understand what will work.

Our findings significantly enhance and expand the current understanding of lipoprotein transfer diversity in Gram-negative bacteria. Furthermore, they reveal that homologous proteins, which serve as potential drug targets, may exhibit varying affinities for antibacterial agents despite having highly similar biological functions. These findings underscore the vast knowledge that remains to be uncovered regarding the diversity of biological systems in bacteria.

## Data Availability

The datasets presented in this study can be found in online repositories. The names of the repository/repositories and accession number(s) can be found below: http://www.wwpdb.org/, 9GTX.
