# Supplementary material for: Structural and functional analysis of the Helicobacter pylori lipoprotein chaperone LolA
Source: Front Microbiol. 2024 Dec 19;15:1512451. doi: 10.3389/fmicb.2024.1512451 (PMC11694511; doi:10.3389/fmicb.2024.1512451)
Supplement: Supplementary file 1 [file Data_Sheet_1.pdf]

Structural and functional analysis of the *Helicobacter pylori* lipoprotein chaperone LolA

Deepika Jaiman[1,2] and Karina Persson[1,2*]

Centre for Microbial Research (UCMR), Umeå University, Umeå, Sweden

[2]Department of Chemistry, Umeå University, Umeå, Sweden.

* Corresponding author:

Karina Persson

E-mail: karina.persson@umu.se

Tel: +46-90-7865926

Running title: Structure of *Helicobacter* LolA

Supplementary figures

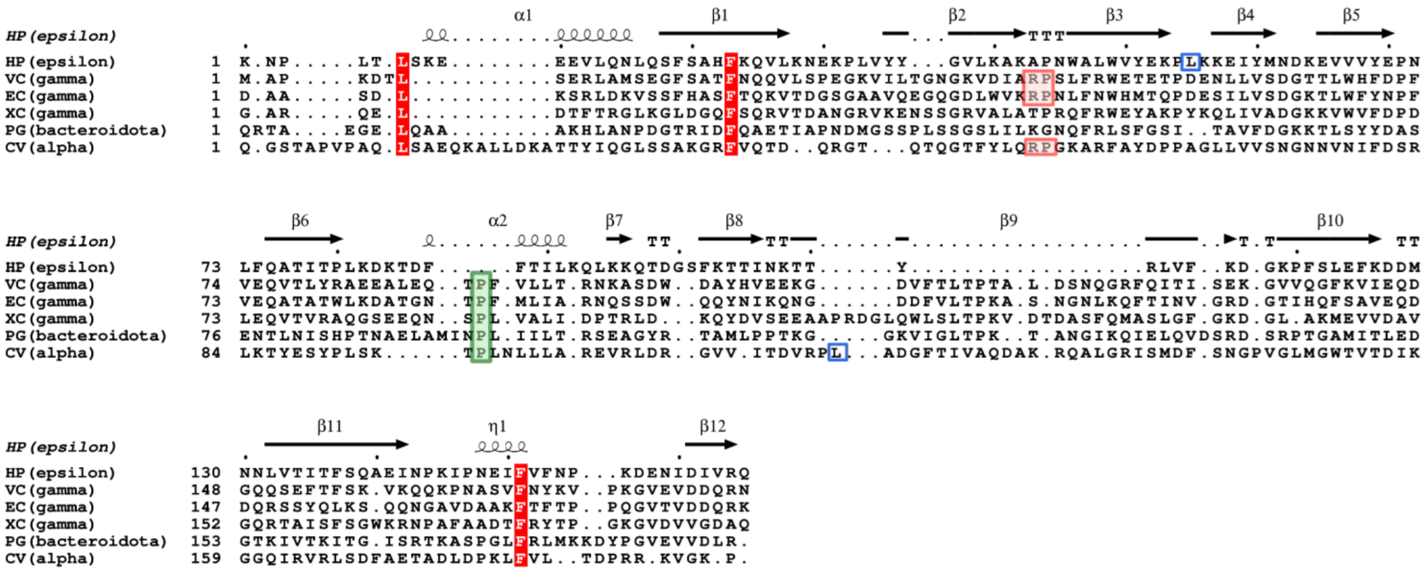

Supplementary figure S1. A structure-based sequence alignment with LolA from different bacteria. (A) *H. pylori* (ε-proteobacteria) on *V. cholerae* (γ-proteobacteria), *E. coli* (γ-proteobacteria), *X.campestris* (γ-proteobacteria), *P. gingivalis* (Bacteroidota) and *C. vibrioides* (α-proteobacteria). All proteins are structurally determined using X-ray crystallography except for *C. vibrioides* which is modelled. Identical residues are shown in red and the Arg-Pro motif, located between β2 and β3 is highlighted in pink. The leucine exposed on the β3β4 loop on LolA-HP and on the β8β9 loop in LolA-CV are marked with a blue box. The proline located between the helices in the binding cleft of all LolA proteins except LolA-HP is highlighted in green.

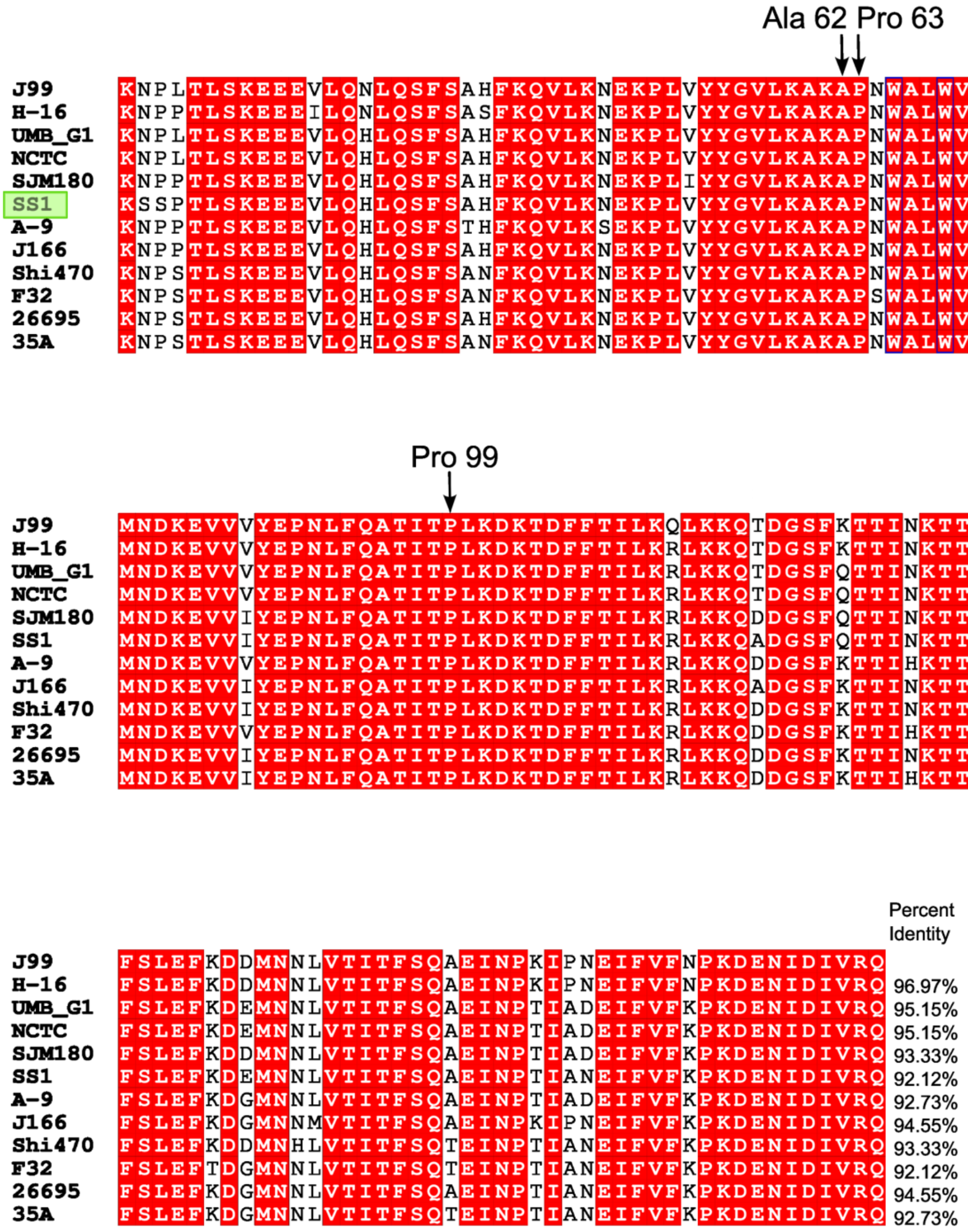

Supplementary figure S2. Sequence alignment of LolA proteins from various *H. pylori* strains.

The alignment demonstrates a high degree of sequence conservation in LolA proteins, with similarities ranging from 92.1% to 97.0%. Key residues (Ala62, Pro63, Leu74, and Pro99) discussed for the LolA *H. pylori* J99 structure are highly conserved, as highlighted in the figure. Notably, in the SS1 strain, Leu74 is substituted with serine in the loop region, as marked by the green arrow.

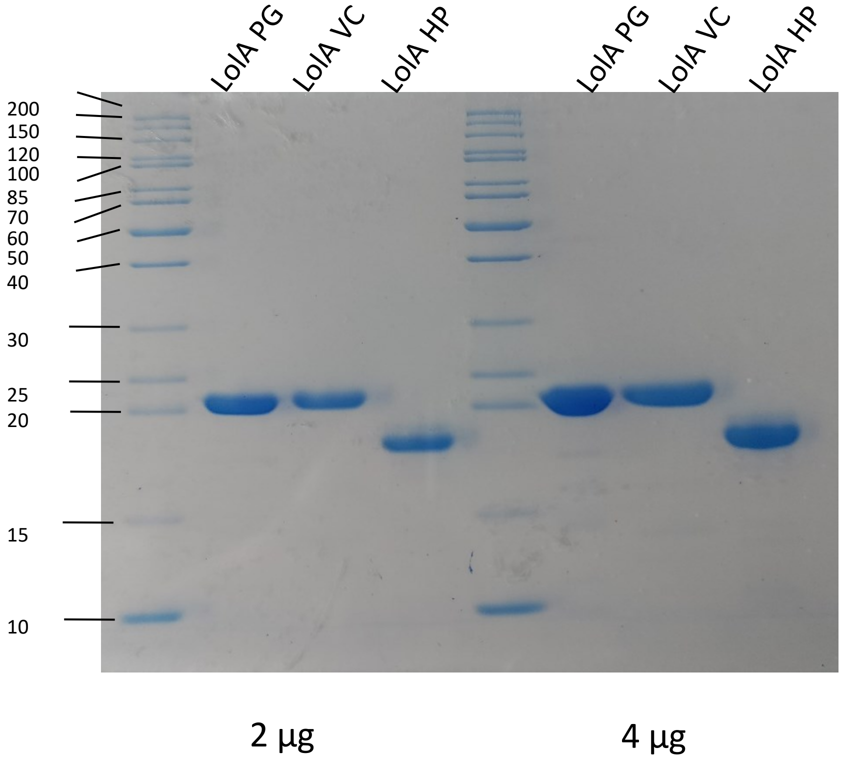

Supplementary figure S3. SDS analysis of the purified proteins. Analysis of LolA-PG, LolA-VC and LolA-HP. The molecular weights, without His-tags, are 20.5, 20.7 and 19.6 kDa respectively. The samples have been analyzed using both 2 µg and 4 µg sample.

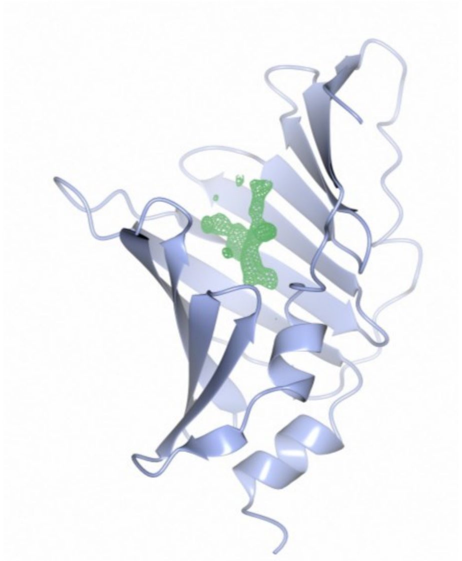

Supplementary figure S4. The binding cleft of LolA-HP contains unmodelled electron density. We hypothesize that the electron density represents a part of pentaerythritol propoxylate from the crystallization solution. The figure depicts the electron density at 3 σ. The figure was prepared with CCP4mg

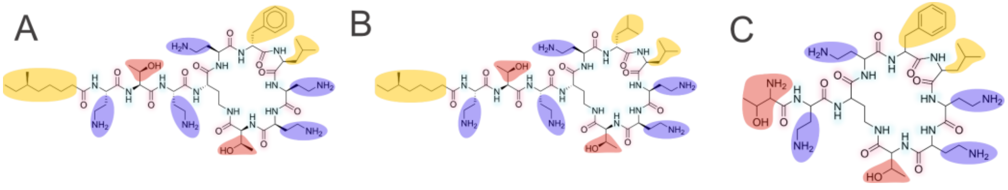

Supplementary figure S5. The substances used in the ITC study. (A) Colistin (Polymyxin E), (B) Polymyxin B and (C) nonapeptide. Hydrophobic side chains are depicted in blue, amino groups in blue and polar groups in red. Colistin and Polymyxin B are lipopeptide antibiotics whereas nonapeptide is a polymyxin derivative that lacks the acyl tail.

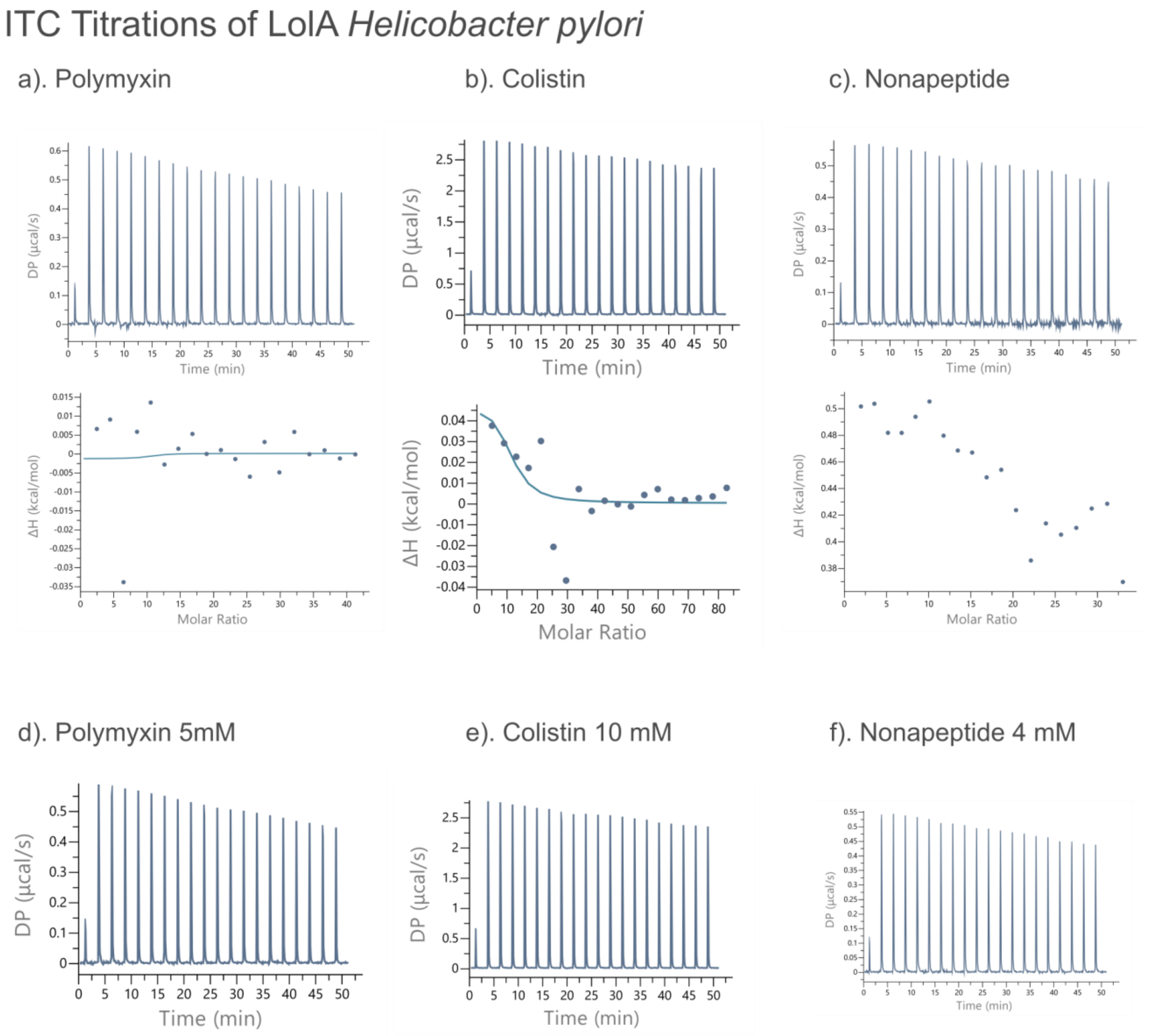

Supplementary figure S6. Representative thermogram for the interaction between LolA-HP and antibiotics measured with ITC. A) polymyxin B) colistin and C) nonapeptide. Final thermograms have been subtracted from heat of injection of ligands to buffer (D, E and F). Values of affinities and thermodynamic parameters of each repeat are given in Table 2.

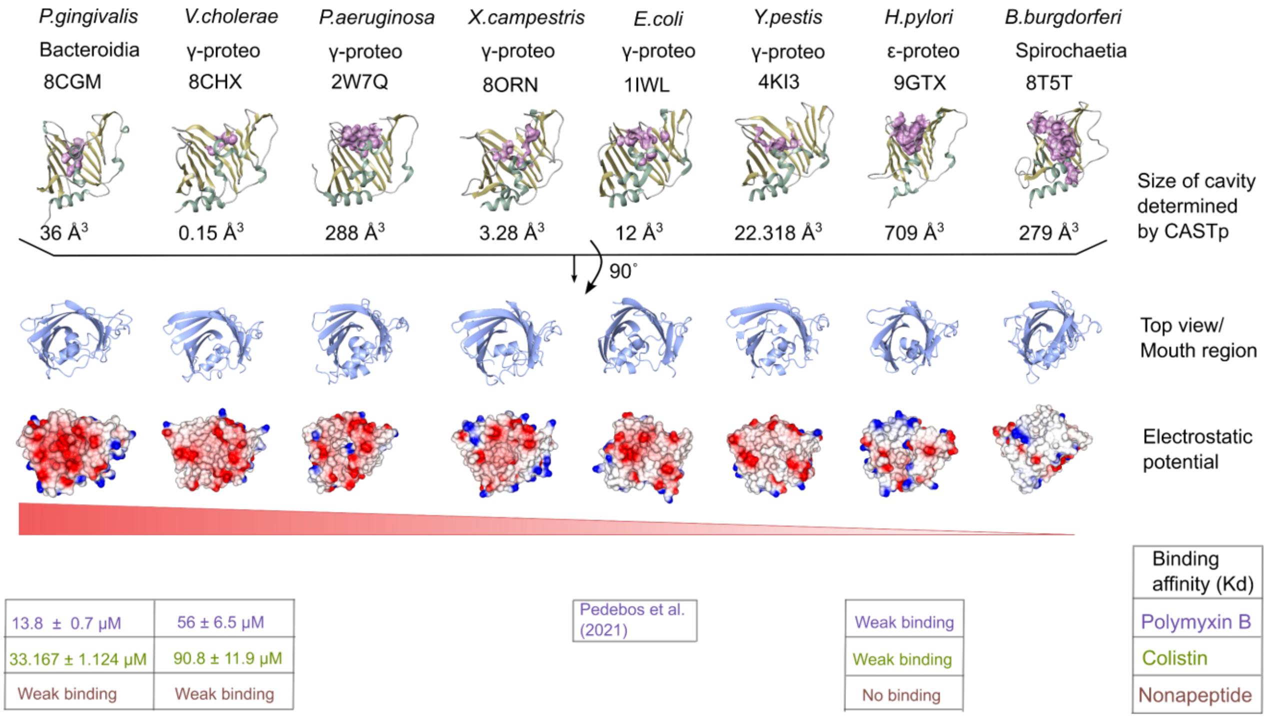

Supplementary figure S7. Electrostatic potential surfaces of LolA proteins from different Gram-negative bacteria. The upper panel shows an overview of the protein, its PDB code, and its binding cavity, calculated by CastP. The view of the protein in the second panel is looking into the binding cleft. The third panel depicts the protein depicted as an electrostatic potential surface. The negative charge of the surfaces decreases from left (*P. gingivalis*) to right (*B. burgdorferi*). The fourth panel presents the experimentally determined Kd values for the interactions between LolA and Polymyxin B, Colistin and Nonapeptide, respectively.

Supplementary Table S1: Primer used in this study

LolA-forward (5’ to 3’)	GTAACCCATGGAAAATCCTTTAACCCTTTCTAAA
LolA-reverse (5’ to 3’)	GTAGAATTCACTGGCGTACAATATCAATATTTTC

Supplementary Table S2: Data processing and refinement statistics

LolA-HP			
**Data collection**		**Refinement**	
Wavelength	0.87313	Resolution (Å)	44.24-2.04
Space group	$P2_1$	No. reflections (work/test)	20693 (2921)
Cell dimensions		R_{work} / R_{free}	0. 202/0.252
a, *b*, *c* (Å)	41.8 65.5 60.8	No. atoms	
α, β, γ (°)	90.0, 99.2, 90.0	Protein	2613
Resolution (Å) *	44.24-2.04 (2.11-2.04)	Ligand/ion	0
R_{merge}	0.132 (1.065)	Water	116
$I / \sigma I$	9.2 (1.8)	*B*-factors (Å^2)	
Completeness (%)	100 (100)	Protein	48.5
Redundancy	6.9 (7.1)	Water	46.8
CC1/2	0.996 (0.494)	R.m.s. deviations	
Molecules in a.u.	2	Bond lengths (Å)	0.007
		Bond angles (°)	0.87
		PDB code	9GTX

*Values in parentheses are for the highest-resolution shell.
